# Use of Red Wine Polyphenols as a Natural Preservative in Health-Promoting Omega-3 Fatty Acids-Enriched Lamb Patties

**DOI:** 10.3390/molecules23123080

**Published:** 2018-11-25

**Authors:** Iria Muíño, Jesús de la Fuente, Concepción Pérez, Elizabeth Apeleo, Cristina Pérez-Santaescolástica, Vicente Cañeque, Sara Lauzurica, Rubén Bermejo-Poza, María Teresa Díaz

**Affiliations:** 1 Departamento de Tecnología de Alimentos. Instituto Nacional de Investigación y Tecnología Agraria y Alimentaria (INIA), Carretera de La Coruña, km. 7, 28040 Madrid, Spain; vcaneque@hotmail.com (V.C.); diaz.teresa@inia.es (M.T.D.); 2 Departamento de Producción Animal, Facultad de Veterinaria, Universidad Complutense de Madrid, Avda. Puerta de Hierro, s/n, 28040 Madrid, Spain; jefuente@vet.ucm.es (J.d.l.F.); elicaz2007@gmail.com (E.A.); cristina.perezsantaescolastica@gmail.com (C.P.-S.); saralauz@ucm.es (S.L.); rbermejop89@gmail.com (R.B.-P.); 3 Sección Departamental Fisiología (Veterinaria), Facultad de Veterinaria, Universidad Complutense de Madrid, Avda. Puerta de Hierro, s/n, 28040 Madrid, Spain; cpmarcos@ucm.es

**Keywords:** polyphenols, -tocopherol, omega-3 fatty acids, meat shelf-life

## Abstract

Meat consumption has been related to a higher risk of heart disease due to its saturated fat content. As a consequence, there has been a growth in research on how to increase unsaturated fat content in meat. However, a high content of unsaturated fat favours the development of oxidative processes. The aim of the study was to evaluate the effectiveness of a red wine extract (RWE) rich in polyphenols (50, 100, and 200 mg gallic acid equivalents/kg meat) as a natural antioxidant in lamb meat patties enriched with omega-3 polyunsaturated fatty acids (*n*-3 PUFA) (100 mg *n*-3 PUFA/100 g meat), compared to using -tocopherol (TOC) (100 mg/kg meat). Adding RWE delayed metmyoglobin formation, lipid oxidation and loss of *n*-3 PUFA relative to controls, while TOC had no effect on preventing meat oxidation. Lamb odour was lower (*p* < 0.01) and odd odour higher (*p* < 0.001) in patties at the highest dose of RWE, compared to controls, but the overall liking score was not affected. The results suggest that RWE could be used as a natural antioxidant in the meat industry, even when *n*-3 PUFA content is high.

## 1. Introduction

One of the main causes of the shift in meat consumption behaviour in recent years is related to health concerns [1]. In this sense, red meat consumption has been linked to the development of chronic diseases mainly due to its saturated fat content, despite the fact that this relationship is not fully justified [2]. As a consequence, the meat industry has developed new meat-based functional foods by including bioactive compounds in their formulations, such as omega-3 polyunsaturated fatty acids (*n*-3 PUFA) [3]. The potential benefits and effects of *n*-3 PUFA, mainly the long chain *n*-3 PUFA (LC *n*-3 PUFA), on human health are widely known, including protective action against cardiovascular diseases, inflammatory diseases, and mental disorders, among others [4]. Furthermore, docosahexaenoic acid (DHA) is deposited in the brain and the retina in humans, having a notable role in the formation and function of the nervous system [5].

However, increasing *n*-3 PUFA content presents a major challenge for the meat industry since they are highly susceptible to lipid oxidation, which eventually compromises both the nutritional and the organoleptic quality of the meat product [6].

Research on the use of naturally-occurring antioxidant compounds, such as tocopherols and plant polyphenols, on the oxidative stability of meat and meat products suggest that they can delay oxidative processes [6]. The antioxidant activity of phenolic compounds depends mainly on their chemical structure and degree of hydroxylation and includes scavenging actions on free radicals, chain-breaking activity, and/or metal chelation properties [7]. Research on the use of plant polyphenols as natural antioxidants in meat products has increased mostly over the last decade and encompasses a great variety of plant sources, i.e., vegetables, fruits, herbs and spices. One of these sources comes from the wine industry, mainly including grape seed and/or peel extracts, but also grape pomace and waste from winery increase lipid stability in poultry, pork, and beef [6].

α-Tocopherol is widely recognised as a chain-breaking antioxidant and it can delay oxidative processes in meat when added to the diet [8]. Nevertheless, when added directly to meat and meat products as a preservative, the results are contradictory, slowing oxidation in some cases, but not in others [9].

The purpose of the present study was to evaluate the effectiveness of adding red wine polyphenols (as natural antioxidants) to health-promoting omega-3-enriched lamb meat, and to compare the effects with using -tocopherol. The study was performed using ground lamb meat stored in high O_2_ modified atmosphere packs (HiOx-MAP), which are commonly used for short-term chilled storage of meat to prevent meat discolouration and microbial spoilage.

## 2. Results 

### 2.1. Instrumental Colour Parameters

Table 1 summarises the results for the colour parameters on lamb patties throughout the storage period (0, 3, 6 and 9 days) for the different treatments (control (CON); red wine extract (RWE): 50 mg GAE/kg muscle (RWE50), 100 mg GAE/kg muscle (RWE100), 200 mg GAE/kg muscle (RWE200); 100 mg -tocopherol/kg muscle (TOC)). An interaction between treatment and storage period was found for L* (*p* ≤ 0.05), a* (*p* ≤ 0.001), hue angle (*p* ≤ 0.001), and MetMb percentage (*p* ≤ 0.001). Lower L* values were found in RWE50 and RWE200 patties compared to TOC patties, and CON and RWE100 patties had intermediate values on day 3 of storage. At 6 days of storage, RWE200 patties presented a lower L* value compared to CON and TOC patties. Differences were found with respect to a* values at the beginning of the storage period (day 0), where RWE100, RWE200 and TOC patties had lower a* values compared to CON patties. The a* values for RWE200 and RWE100 patties were more consistent during the entire storage period than for RWE50, TOC and CON patties, where they decreased more sharply. Regarding hue angle, lower values were found in RWE100 and RWE200 patties compared to CON and TOC patties at days 6 and 9 of storage period. With respect to MetMb percentage, RWE and TOC patties had a lower rate of MetMb formation than CON patties at 6 days of storage period, and RWE patties had lower percentage of MetMb compared to CON, and TOC patties at the end of storage (day 9). 

### 2.2. Lipid and Protein Oxidation

**Figure 1:**
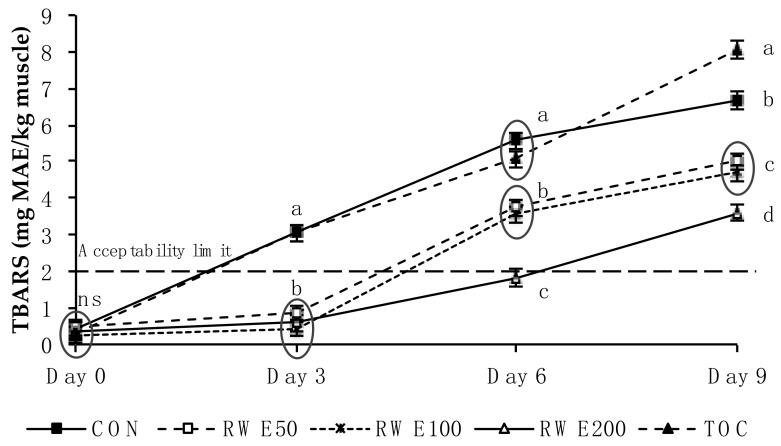
Lipid oxidation (TBARS) measured in *n*-3-enriched lamb meat patties over the 9-day storage period in high oxygen modified atmosphere packs (HiOx-MAP), containing different levels of antioxidants. CON: control without antioxidant; RWE50: 50 mg GAE/kg muscle; RWE100: 100 mg GAE/kg muscle; RWE200: 200 mg GAE/kg muscle; TOC: 100 mg -tocopherol/kg muscle. Data are expressed as mean value ± standard error of three replicates in duplicate. Different letters (a–d) mean significant differences among treatments (*p* ≤ 0.05).

An interaction between treatment and storage period (*p* ≤ 0.001) was found for lipid oxidation (TBARS values) (Figure 1). From day 3 onwards, TBARS values in CON and TOC patties increased greatly in comparison to RWE patties, and RWE200 patties showed a less TBARS content on day 6 and 9 of storage period with respect to RWE50 and RWE100 patties.

**Figure 2:**
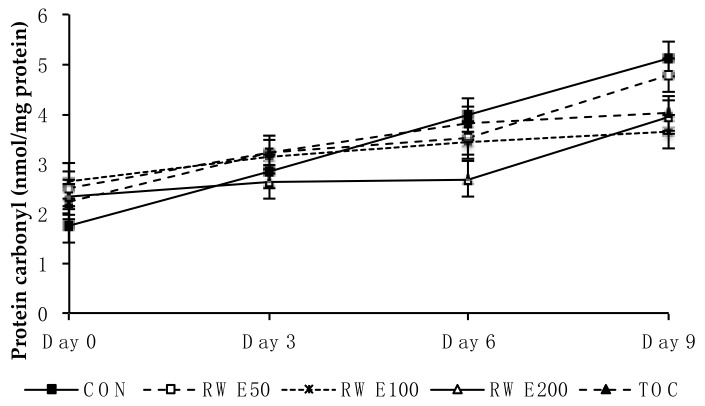
Protein oxidation (carbonyl groups) measured in *n*-3-enriched lamb meat patties over the 9-day storage period in high oxygen modified atmosphere packs (HiOx-MAP), containing different levels of antioxidants. CON: control without antioxidant; RWE50: 50 mg GAE/kg muscle; RWE100: 100 mg GAE/kg muscle; RWE200: 200 mg GAE/kg muscle; TOC: 100 mg -tocopherol/kg muscle. Data are expressed as mean value ± standard error of three replicates in triplicate.

In relation to protein oxidation, there were no differences between treatments on protein carbonyl content (Figure 2), which increased (*p* ≤ 0.001) during the storage period in all types of patties. 

### 2.3. Long Chain (LC) n-3 PUFA Content

In the present study, a mean value of 91.6 mg eicosapentaenoic acid (EPA) + docosahexaenoic acid (DHA)/100 g meat was obtained, with no differences between treatments at 0 days (Table 2). There was an interaction between treatment and storage period (*p* ≤ 0.01). The content of LC *n*-3 PUFA decreased in all types of patties over the storage period, nevertheless this decrease was less noticeable manner in RWE200 patties, which had a higher content than CON patties at 9 days. 

### 2.4. Sensory Evaluation

Data on the sensory analysis of cooked lamb patties throughout the storage period are presented in Table 3. In general, sensory attributes were affected by storage period and minor differences were found among treatments. There were no significant interactions between treatment and storage period for any of the attributes studied. 

Lamb and odd odour decreased during storage and both were affected by treatment. The lowest value for lamb odour and the highest score for odd odour were found in RWE200 patties. Fish odour increased in all types of patties during the storage period in a more noticeable manner than rancid odour. There were no differences for fatty and rancid flavours, neither during storage period nor between treatments. Lamb and odd flavour decreased and fish flavour increased over the storage period. The texture of lamb patties was affected by storage period, with juiciness decreasing and chewiness increasing on day 3 of storage. The overall liking of cooked lamb patties decreased at 3 days of storage and it was not affected by any of the treatments studied. 

## 3. Discussion

Appearance and, in particular meat colour, is the main attribute that influences consumer purchase decisions, since they associate brown colour with a loss of meat freshness and with wholesomeness [10]. According to MacDougall [11] an increase of L* values could be related to an increase on light scattering produced by changes in the structure of muscle proteins as a consequence of the oxidative processes, rather than a result of changes in the myoglobin redox state [12]. Therefore, a lower L* value on RWE200 patties (Table 1) could be associated with less intense oxidation. Lower a* values were found at the beginning of the storage period for RWE100, RWE200, and TOC patties in relation to CON patties (Table 1), which may be related to the colour of the additives used, since the addition of different extracts can change the product’s natural colour. For instance, the addition of grape seed flour to beef frankfurters resulted in a decrease in L*, a* and b* values [13], and cooked chicken meatballs with a grape extract showed lower L* and b* values than controls [14]. Despite this, both RWE100 and RWE200 patties showed a more stable and higher a* value during the storage period, maintaining a better colour from the point of view of consumers [15]. Hue angle has been proposed as a good indicator of discolouration in meat. Increasing values of hue angle indicate discolouration of meat as a consequence of a decrease in a* and an increase of MetMb percentage [16] on the meat surface, that is, ranging from red to brown as a result of myoglobin oxidation [10]. Therefore, the colour of RWE100 and RWE200 patties was more stable since changes in the hue angle were less noticeable throughout storage. Indeed, hue angle did not change at all in RWE200 throughout storage (Table 1). In accordance with these results, the MetMb proportion in RWE100 and RWE200 patties was lower than for CON patties from day 6 onward (Table 1); indicating that the oxidation of red oxymyoglobin to brownish metmyoglobin was less pronounced. It has been reported that meat with greater than 40% of MetMb are downgraded by trained judges and consumer panels [17]. Taking into account this acceptability limit, CON patties would be rejected at 6 days and consumers would reject TOC patties at 9 days while RWE patties might still be acceptable. Therefore, the results obtained point to the potential of using RWE to prevent meat discolouration. In line with our findings, the use of a white grape extract (500 mg/kg meat) in beef patties stored in HiOx-MAP maintained a redder colour at the end of storage period (9 days) in comparison with control samples [18]. Similarly, the use of a grape seed extract (250 mg/kg meat) in ground beef enriched with n-3 PUFA and stored in aerobic conditions for up to 6 days, resulted in higher a* values compared to controls [19]. However, in the latter study the addition of grape seed extract was not able to slow down MetMb formation when ground meat was enriched with n-3 PUFA. Regarding the effect of α-tocopherol in preventing meat discolouration, Lee et al. [9] found greater a* values and lower hue angle values when adding rosemary extract to *n*-3 oil fortified beef patties than when using a mixture of tocopherol isomers, similar to the results obtained in the present study.

In relation to lipid oxidation, adding RWE to lamb patties delayed lipid oxidation at all time points in comparison to CON and TOC patties, with RWE200 patties not exceeding the threshold value of 2 mg MAE/kg of muscle for rancidity perception [20] during the first 6 days of storage. Results obtained using RWE are in agreement with those reported by Gómez et al. [19] using 250 mg grape seed extract/kg meat in ground beef enriched with *n*-3 PUFA and stored in aerobic packs at 2 °C for 6 days in an illuminated retail display, where a delaying on lipid oxidation processes was observed. In line with our results, Lee et al. [9] did not find significant differences relative to controls with respect to TBARS values when adding 0.03% mixed tocopherol isomers (based on fat content) to ground beef enriched with LC *n*-3 PUFA (500 mg fish oil/110 g meat) stored in aerobic packs during 6 days at 4 °C. Other authors have found a slight reduction of lipid oxidation when a mixture of tocopherols was used in meat products, however this effect was clearly weaker compared to the use of plant extracts such as rosemary extracts [21]. The undisputed efficiency of α-tocopherol as a chain-breaking antioxidant is due to its location within the cellular membrane, in close proximity to the phospholipid fatty acids, which also accounts for membrane integrity [22]. The fact that, in the present study, α-tocopherol was added directly to meat and was not located in its natural position, together with the disruption of muscle membrane by mincing that releases haeme and nonhaeme iron (which acts as catalyst for lipid oxidation), could lead to the inability of α-tocopherol to associate with muscle phospholipids and protect them against oxidation [23], thus failing to prevent TBARS formation. Moreover, the content of LC *n*-3 PUFA (highly susceptible to oxidation) present in the meat and/or a high proportion of oxygen (HiOx-MAP) could make the dose used insufficient to slow down lipid oxidation. 

Contrary to the results obtained regarding lipid oxidation, the addition of RWE to lamb patties did not prevent protein oxidation at any time during the storage period (Figure 2). Also, no differences were found by Jongberg et al. [18] for protein carbonyl content between beef patties added with white grape extract (500 ppm of extract in the meat) and controls, during a 9-day storage period in HiOx-MAP at 4 °C. 

Regarding LC *n*-3 PUFA content and in accordance with the Commission Regulation (UE) [24], each kind of lamb patty may be considered as a “high in omega-3 fatty acids” product during the first half of storage period (day 3), since they had more than 80 mg EPA+DHA/100 g product. In fact, RWE200 patties would be still labelled as a “source of omega-3 fatty acids”, due to the presence of more than 40 mg EPA+DHA/100g product at the end of storage (day 9). These results could be related to those explained early regarding TBARS values where RWE200 patties showed the highest effectiveness in retarding lipid oxidation (Figure 1). In the same way as for TBARS values, no antioxidant activity was observed in TOC patties for preventing loss of LC *n*-3 PUFA. 

In relation to the results of the sensory evaluation (Table 3), the highest score for odd odour found in RWE200 patties could be the result of the tannic odour of the extract used, which may have covered up the lamb odour in the same group (which was in turn lowest for this attribute). Rancid odour increased over the storage period, probably due increased lipid oxidation, which resulted in development of off-odours [6]. However, no differences were found among treatments despite the fact that at 3 days of storage, clear differences were observed regarding TBARS values (Figure 1). It is worth noting that panellists scored very low rancid odour for every type of lamb patty, which may partly explain why there were not significant differences for this attribute. Similar to our results, adding 300 mg/kg grape seed extract to beef patties did not affect rancid odour development [25]. The fact that, in the current study, LC *n*-3 PUFA were added to meat directly as fish oil could increase fish odour and flavour during the storage period, which was also observed in PUFA-fortified foods with added fish oils [26]. Both attributes of meat texture (juiciness and chewiness) changed over the storage period and might be related to the increase of protein carbonyl groups mentioned above, which took place at 3 days of storage in the five treatments (Figure 2), since texture can be affected by protein oxidation [27]. Overall liking was similar among treatments, implying that lamb odour and odd odour did not influence the general acceptability of lamb patties, and that RWE could be used effectively by the meat industry. 

The European market for functional foods is still smaller than in other parts of the world, such as Japan and US, mainly due to regulation patterns and consumer opinion regarding these types of products [28]. Nevertheless, research regarding future trends in meat consumption in Spain has revealed that, although the consumption of functional foods is low, there will be an increase in demand for modified lipid profile meat products in the coming years [29]. In fact, consumers prefer beef meat enriched with omega-3 fatty acids as long as it does not compromise sensory attributes [30]. 

Recommendations for the suggested daily intake levels of EPA and DHA vary globally depending on the regional health advisory organisations and authorities [31]. According to the EFSA Panel on Dietetic Products, Nutrition, and Allergies [4], 250 mg/day of EPA plus DHA has been proposed as an adequate intake for healthy adults. Taking this data into consideration, 3-day-old patties from the present study would cover about 37% of the daily-recommended intake and RWE200 patties would still provide 25% of this intake after 9 days of storage period (Table 2). Moreover, RWE increased the shelf life of lamb patties from an oxidative point of view, keeping RWE200 lamb patties below the acceptability limit for rancidity from lipid oxidation (<2 mg MAE/kg muscle; [20]) until 6 days of storage (Figure 1) and preventing meat discolouration in all RWE patties in terms of MetMb percentage (<40%; [17]) during the entire storage period (9 days). 

Nonetheless, polyphenols present in RWE may have an activity beyond their antioxidant function as a food preservative, since their consumption has been related to biological health actions such as anticarcinogenic, anti-inflammatory, antiatherogenic, and cardioprotective effects [32]. The main antioxidant effect of red wine polyphenols seems to take place at the gastrointestinal level, preventing oxidation of lipids and other important dietary constituents such as vitamin E and, consequently, reducing the levels of lipid peroxides in plasma after eating red meat [33], which in turn underlines the importance of a PUFA-enriched meal. 

Despite the fact that in the present study the addition of α-tocopherol did not affect the oxidative stability of lamb patties, it would be interesting to evaluate a combination of RWE and α-tocopherol in future studies, for several reasons. On the one hand, when α-tocopherol acts as an antioxidant by donating its phenolic hydrogen, a tocopheroxyl radical is formed [22]. Red wine polyphenols could reduce this radical to regenerate α-tocopherol, thereby exerting a synergetic effect [34]. On the other hand, with this recycling of α-tocopherol, it may be possible to use different doses of RWE in order to reduce the odd odour detected by the sensory panel, which would be the main drawback in the present study for achieving a marketable health-promoting meat product.

## 4. Materials and Methods 

### 4.1. Preparation, Packaging, and Storage of Lamb Patties

Lamb legs were purchased from a local supermarket and transported under refrigeration (2 °C) to the pilot plant (INIA, Madrid, Spain). The quadriceps femoris muscles were dissected and connective tissues and subcutaneous fat were removed. Muscle were cut into small pieces and minced twice, once through a 5 mm plate (PM-70 model, Mainca, Granollers, Spain) and after through a 3 mm plate. Deodorised fish oil (Algatrium plus, Brudy Technology, Barcelona, Spain) was added to minced meat to produce approximately 100 mg LC *n*-3 PUFA/100 g of meat. Five batches of ground lamb meat were manufactured: 100 mL of cold water was added to the control group batch (CON patties); increasing concentrations of red wine extract (RWE) (Provinols^TM^, Seppic, S.A., Paris, France) diluted in cold water (final volume 100 mL) were added to three other batches (RWE patties) to obtain the final total phenol contents of 50, 100, and 200 mg GAE/kg meat (RWE50, RWE100, and RWE200, respectively); and 100 mg α-tocopherol (Panreac, Barcelona, Spain)/kg meat in cold water (final volume 100 mL) were added to the last batch (TOC patties). The detailed characteristics of RWE are described elsewhere [8].

Minced lamb meat was thoroughly mixed in a food mixer (RM-20, Mainca, Granollers, Spain) and lamb patties of 100 g with 10 cm diameter and 1 cm thick were formed in a burger-maker. Some lamb patties were directly vacuum-packed in metallic polyester pouches (PET/MET+PE, Sacoliva SL^®^, Barcelona, Spain) of low O_2_ permeability (<1.5 cc/m^2^/24 h) and low water vapour transmission rate (<1.5 mg/m^2^/24 h) and frozen at −20 °C remaining as 0-day samples. The rest of the lamb patties were placed in high oxygen modified atmosphere packaging (HiOx-MAP, 70% O_2_/30% CO_2_) using a packing machine (EV-15-1-CD-SC, Tecnotrip S.A., Barcelona, Spain) and stored in the dark at 4 °C for 3, 6, or 9 days. The pouches used for MAP (PA/PE, Sacoliva S.L.^®^, Barcelona, Spain) were composed of 150 µm polyamide/polyethylene (50/100), with low gas permeability (18 cc/m^2^/24 h O_2_ at 23 °C, 55 cc/m^2^/24 h CO_2_, and 4.0 cc/m^2^/24 h at 23 °C) and low water vapour transmission rate (1.8 g/m^2^/24 h at 23 °C and 85% relative humidity). After each storage time, lamb patties were vacuum-packaged and frozen as explained above, until required for analysis. 

### 4.2. Instrumental Colour Measurements 

Colour parameters were measured in the CIE L*a*b* colour space (measured area diameter of 8 mm, D_65_, observer angle 10°) using a CM-2006d spectrophotometer (Minolta Camera Co., Osaka, Japan). Three measurements were taken on three non-overlapping zones of the sample at each storage time (0, 3, 6, and 9 days) and mean values were calculated. The lightness (L*), redness (a*) and yellowness (b*) were recorded, and hue angle was calculated as tan^−1^ (b*/a*). The relative proportion of metmyoglobin (MetMb) was calculated according to Krzywicki [35], taking the achromatic absorption of lamb meat at 690 nm [36]. 

### 4.3. Measurement of Lipid And Protein Oxidation

Lipid oxidation was assessed by thiobarbituric acid reactive substances (TBARS) values, in duplicate at each storage time (days 0, 3, 6 and 9), following the method proposed by Maraschiello et al. [37]. Results were expressed as mg malonaldehyde equivalents (MAE)/kg meat.

Protein oxidation was assayed according to the method of derivatisation with 2,4-dinitrophenylhydrazine (DNPH) described by Ganhão et al. [38] the formation rate of carbonyl groups was measured in triplicate over the storage period (on days 0, 3, 6, and 9). Results were expressed as nmol carbonyl groups/mg protein.

### 4.4. Fatty Acid Analysis

Fatty acid methyl esters (FAMES) of freeze-dried lamb patties were formed in duplicate according to the method proposed by Lee et al. [39] in samples from 0, 3, 6, and 9 days. Tridecanoic acid (13:0) was added as an internal standard (Sigma-Aldrich Co., St. Louis, MO, USA). FAMES were analysed by gas chromatography with flame ionization detection (Perkin-Elmer Autosystem-1:A, MA, USA). The technical characteristics of the chromatography method employed for fatty acid characterization have been previously reported [8]. Individual FAMES were identified by comparing their retention times with those from a standard FAME mixture (Sigma-Aldrich Co., St. Louis, USA). Results were expressed as mg FAMES/100 g meat.

### 4.5. Sensory Analysis of Cooked Patties

Sensory evaluation was performed in lamb patties after 0 and 3 days of storage. The sensory panel was trained as previously reported by Muíño et al. [8]. Briefly, eight panellists were trained in the sensory profiling of lamb meat and in the specific lamb, fat, and oxidised odour and flavour. Additionally, the sensory panel was trained to assess fish odour and flavour. The sensory descriptors assessed by the panellists were odour (lamb, rancid, fish, odd), flavour (lamb, fatty, rancid, fish, odd), texture (juiciness, chewiness), and overall liking. 

Patties were thawed overnight at 4 °C, wrapped in aluminium foil and cooked in a pre-heated domestic oven (Unox, Vigodarzere, Italy) at 175 °C, until an internal temperature of 70 °C was reached. The internal temperature was measured by a thermocouple probe (K-type thermocouple). After cooking, samples were cut into portions of 2 cm^2^ and wrapped in aluminium foil. Each sample was labelled with a single random three-digit code and placed in a heated incubator until sensory analysis. Panellists assessed ten samples per session: two storage periods (0 and 3 days) for each of the five treatments, using an unstructured 100-mm line scale, anchored at each end. Panel members evaluated the samples under red light conditions. Unsalted breadsticks and room temperature water were provided to the panellists for cleaning the palate between samples.

### 4.6. Statistical Analysis

Data were analysed using the 9.1.2 Statistical Analysis System package (SAS Institute, Cary, NC, USA). Instrumental colour parameters, TBARS values, protein carbonyl groups, fatty acid composition and sensory analysis were analysed by a two-way analysis of variance using the GLM procedure with treatment and storage period as main factors, and the interaction between both factors was included in the model. For sensory analysis, the effect of the panellist and the session were included in the model. When significant differences were observed between main factors and when the interaction between both factors (treatment x storage period) was significant, a Dunn-Šidak test was performed for mean comparisons (*p* ≤ 0.05).

## 5. Conclusions

Adding red wine extract to lamb meat patties enriched in omega-3 fatty acids delayed colour deterioration in terms of metmyoglobin formation during a 9-day storage period. The highest dose of extract (200 mg gallic acid equivalent/ kg meat) kept lamb patties within consumers’ acceptability for lipid oxidation during 6 days of storage. In contrast, α-tocopherol was less effective at promoting oxidative stability of omega-3 enriched lamb patties. The overall acceptability of lamb patties was not affected by adding the red wine extract, despite the fact that panellists reported an odd odour. The designed RWE lamb meat patties would be labelled as “high in omega-3 fatty acids” products and they would be still sold as “source of omega-3 fatty acids” products at the end of storage period, covering from 16 to 25% of the daily-recommended intake for long chain omega-3 fatty acids. Furthermore, consumer might benefit from the potential antioxidant effect of RWE in the organism after consumption. 

## Figures and Tables

**Table 1:** Instrumental colour parameters measured in *n*-3-enriched lamb meat patties over the 9-day storage period (SP) in high oxygen modified atmosphere packs (HiOx-MAP), containing different levels of antioxidants.

	SP	Treatment (T)					SEM	Significance		
	(days)	CON	RWE50	RWE100	RWE200	TOC		T	SP	TxSP
L*							2.14	***	ns	*
	0	45.3	45.8	45.4	43.6	45.4				
	3	45.4 ^wx^	41.8^x^	44.1 ^wx^	41.9^x^	48.3 ^w^				
	6	48.3^w^	45.5 ^w^	43.2 ^wx^	39.0^x^	46.6 ^w^				
	9	47.1	47.1	43.2	42.1	46.3				
a*							1.02	***	***	***
	0	16.6 ^a,w^	14.1 ^a,wx^	13.3 ^a,xy^	12.5 ^a,xy^	10.9 ^a,y^				
	3	11.6 ^b^	10.6^b^	10.4 ^b^	10.2 ^ab^	9.53 ^ab^				
	6	6.64 ^c,x^	8.88^bc,wx^	9.78 ^b,w^	9.17 ^ab,w^	7.32 ^bc,wx^				
	9	6.33 ^c^	6.90 ^c^	7.65 ^b^	7.82 ^b^	5.35 ^c^				
Hue angle							2.38	***	***	***
	0	52.5 ^b^	54.9 ^b^	55.9 ^b^	57.1 ^a^	58.4 ^b^				
	3	56.6 ^b^	60.1 ^ab^	57.5 ^ab^	56.8 ^a^	60.4 ^ab^				
	6	67.1 ^a,w^	62.9 ^a,wx^	57.0 ^ab,x^	59.4 ^a,x^	66.9 ^a,w^				
	9	71.0 ^a,w^	66.1 ^a,wx^	63.0 ^a,x^	60.9 ^a,x^	71.4 ^a,w^				
MetMb							2.31	***	***	***
(%)	0	16.5 ^d^	16.8 ^b^	18.0 ^c^	19.4 ^c^	15.6 ^d^				
	3	26.7 ^c,x^	34.1 ^a,w^	26.2 ^b,x^	25.9 ^b,x^	26.7 ^c,x^				
	6	42.5 ^b,w^	30.5 ^a,x^	31.1 ^ab,x^	30.6 ^ab,x^	34.4 ^b,x^				
	9	49.4 ^a,x^	36.5 ^ab,y^	34.8 ^a,y^	34.5 ^a,y^	59.1 ^a,w^				

Data are expressed as the mean of three replicates in triplicate. CON: control without antioxidant; RWE50: 50 mg GAE/kg muscle; RWE100: 100 mg GAE/kg muscle; RWE200: 200 mg GAE/kg muscle; TOC: 100 mg α-tocopherol/kg muscle; RWE: red wine extract; GAE: gallic acid equivalents; SEM: standard error of the mean. Significance: ns: *p* > 0.05; *: *p* ≤ 0.05; ***: *p* ≤ 0.001. ^a,b,c,d^ Different superscript letters within the same column indicate significant difference (*p* ≤ 0.05). ^w,x,y^ Different superscript letters within the same row indicate significant difference (*p* ≤ 0.05).

**Table 2:** Long chain *n*-3 PUFA (EPA + DHA) measured in *n*-3-enriched lamb meat patties over 9-day storage period (SP) in high oxygen modified atmosphere packs (HiOx-MAP), containing different levels of antioxidants.

	SP	Treatment (T)					SEM	Significance		
	(days)	CON	RWE50	RWE100	RWE200	TOC		T	SP	TxSP
EPA+DHA							157.3	ns	***	**
(mg/100 g	0	95.2 ^a^	99.8 ^a^	89.7 ^a^	95.7 ^a^	77.8 ^a^				
meat)	3	91.8 ^a^	95.6 ^a^	110.0 ^a^	80.5 ^ab^	82.7 ^a^				
	6	72.6 ^a^	76.4 ^a^	57.3 ^b^	69.6 ^ab^	63.3 ^a^				
	9	21.5 ^b,w^	39.6 ^b,wx^	43.6 ^b,wx^	62.5 ^b,x^	31.0 ^b,wx^				

Data are expressed as the mean of three replicates in duplicate. EPA: eicosapentaenoic acid; DHA: docosahexaenoic acid; CON: control without antioxidant; RWE50: 50 mg GAE/kg muscle; RWE100: 100 mg GAE/kg muscle; RWE200: 200 mg GAE/kg muscle; TOC: 100 mg -tocopherol/kg muscle; RWE: red wine extract; GAE: gallic acid equivalents; SEM: standard error of the mean. Significance: ns: *p* > 0.05; **: *p* ≤ 0.01; ***: *p* ≤ 0.001. ^a,b^ Different superscript letters within the same column indicate significant difference (*p* ≤ 0.05). ^w,x^ Different superscript letters within the same row indicate significant difference (*p* ≤ 0.05).

**Table 3:** Sensory evaluation of cooked *n*-3 enriched lamb meat patties over the 9-day storage period (SP) in high oxygen modified atmosphere packs (HiOx-MAP), containing different levels of antioxidants.

	SP	Treatment (T)						*SEM*	Significance		
	(days)	CON	RWE50	RWE100	RWE200	TOC	Mean		T	SP	TxSP
Lamb odour								15.5	**	***	ns
	0	35.1	33.0	24.7	18.8	34.4	29.2				
	3	23.1	21.9	19.6	16.9	20.9	20.5				
Mean		29.1^w^	27.4^w^	22.1^wx^	17.8^x^	27.7^w^					
Rancid odour								3.28	ns	*	ns
	0	0.39	0.51	1.05	0.64	0.52	0.62				
	3	1.68	0.65	0.77	2.92	2.37	1.68				
Mean		1.04	0.58	0.91	1.78	1.44					
Fish odour								17.0	ns	***	ns
	0	1.00	0.00	4.19	2.28	4.58	2.39				
	3	28.7	25.3	11.3	18.5	23.8	21.5				
Mean		14.9	12.6	7.74	10.4	14.2					
Odd odour								15.1	***	*	ns
	0	8.07	11.3	20.5	28.9	13.4	16.4				
	3	12.9	7.94	15.7	20.6	7.50	12.9				
Mean		10.5^x^	9.61^x^	18.1^wx^	24.8^w^	10.4^x^					
Lamb flavour								14.9	ns	***	ns
	0	37.0	33.0	31.2	25.2	36.8	32.6				
	3	18.1	18.6	22.8	21.1	24.2	21.0				
Mean		27.5	25.8	27.0	23.2	30.5					
Fatty flavour								5.68	ns	ns	ns
	0	4.16	3.85	2.13	1.38	0.00	2.30				
	3	3.80	1.59	0.32	4.26	4.13	2.82				
*Mean*		3.98	2.72	1.22	2.82	2.05					
Rancid flavour								6.79	ns	ns	ns
	0	0.00	3.22	2.00	0.23	0.00	0.98				
	3	2.09	1.46	0.00	5.74	2.82	2.28				
Mean		0.81	2.34	0.64	2.99	1.36					
Fish flavour								18.5	ns	***	ns
	0	2.24	0.00	5.35	0.43	5.58	2.57				
	3	31.0	31.3	18.5	26.1	29.6	27.3				
Mean		16.6	15.3	11.9	13.3	17.6					
Odd flavour								15.7	ns	***	ns
	0	16.4	31.5	23.3	32.2	21.9	25.1				
	3	17.5	16.0	14.3	16.2	8.98	14.6				
Mean		17.0	23.7	18.8	24.2	15.4					
Juiciness								17.5	*	***	ns
	0	47.5	41.3	49.6	59.7	47.1	49.0				
	3	34.8	36.8	38.9	41.2	34.5	37.2				
Mean		41.2^wx^	39.1^x^	44.2^wx^	50.4^w^	40.8^wx^					
Chewiness								15.7	ns	**	ns
	0	26.1	24.6	21.7	15.6	20.7	21.8				
	3	30.4	22.8	28.1	26.4	27.4	27.0				
Mean		28.3	23.7	24.9	21.0	24.1					
Overall liking								18.2	ns	***	ns
	0	51.0	47.6	55.0	48.1	49.2	50.2				
	3	30.3	28.2	29.5	32.1	30.8	30.2				
Mean		40.7	37.9	42.3	40.1	40.0					

Sensory scale: 100-mm unstructured scale. CON: control without antioxidant; RWE50: 50 mg GAE/kg muscle; RWE100: 100 mg GAE/kg muscle; RWE200: 200 mg GAE/kg muscle; TOC: 100 mg -tocopherol/kg muscle; RWE: red wine extract; GAE: gallic acid equivalents; SEM: standard error of the mean. Significance: ns: *p* > 0.05; *: *p* ≤ 0.05; **: *p* ≤ 0.01; ***: *p* ≤ 0.001. ^w,x^ Different superscript letters within the same row indicate significant difference (*p* ≤ 0.05).
